# Mesenchymal stromal cells from human umbilical cord prevent the development of lung fibrosis in immunocompetent mice

**DOI:** 10.1371/journal.pone.0196048

**Published:** 2018-06-01

**Authors:** Gianluca Moroncini, Chiara Paolini, Fiorenza Orlando, Chiara Capelli, Antonella Grieco, Cecilia Tonnini, Silvia Agarbati, Eleonora Mondini, Stefania Saccomanno, Gaia Goteri, Silvia Svegliati Baroni, Mauro Provinciali, Martino Introna, Nicoletta Del Papa, Armando Gabrielli

**Affiliations:** 1 Dipartimento di Scienze Cliniche e Molecolari, Università Politecnica delle Marche, Ancona, Italy; 2 Centro di Tecnologie Avanzate nell’Invecchiamento, IRCCS-INRCA, Ancona, Italy; 3 UOS Centro di Terapia Cellulare "G. Lanzani", A.S.S.T. Papa Giovanni XXIII, Bergamo, Italy; 4 Dipartimento di Medicina Sperimentale e Clinica, Università Politecnica delle Marche, Ancona, Italy; 5 AnatomiaPatologica, Dipartimento di Scienze Biomediche s e Sanità Pubblica, Università Politecnica delle Marche, Ancona, Italy; 6 UOC Day Hospital di Reumatologia, Dipartimento di Reumatologia, ASST G. Pini-CTO, Milano, Italy; Centre National de la Recherche Scientifique, FRANCE

## Abstract

Lung fibrosis is a severe condition resulting from several interstial lung diseases (ILD) with different etiologies. Current therapy of ILD, especially those associated with connective tissue diseases, is rather limited and new anti-fibrotic strategies are needed. In this study, we investigated the anti-fibrotic activity in vivo of human mesenchymal stromal cells obtained from whole umbilical cord (hUC-MSC). Adult immunocompetent C57BL/6 mice (n. = 8 for each experimental condition) were injected intravenously with hUC-MSC (n. = 2.5 × 10^5^) twice, 24 hours and 7 days after endotracheal injection of bleomycin. Upon sacrifice at days 8, 14, 21, collagen content, inflammatory cytokine profile, and hUC-MSC presence in explanted lung tissue were analyzed. Systemic administration of a double dose of hUC-MSC significantly reduced bleomycin-induced lung injury (inflammation and fibrosis) in mice through a selective inhibition of the IL6-IL10-TGFβ axis involving lung M2 macrophages. Only few hUC-MSC were detected from explanted lungs, suggesting a “hit and run” mechanism of action of this cellular therapy. Our data indicate that hUC-MSC possess strong in vivo anti-fibrotic activity in a mouse model resembling an immunocompetent human subject affected by inflammatory ILD, providing proof of concept for ad-hoc clinical trials.

## Introduction

Mesenchymal stromal cells (MSC) are adult multipotent cells which can be isolated from bone marrow (BM), adipose tissue, umbilical cord (UC), amniotic membrane and other tissues, and besides their ability of differentiating into different cell lineages have raised growing interest because of their immunomodulatory properties[1, 2]. By virtue of these properties, they are currently widely employed in the field of autoimmune rheumatic diseases[3, 4] and fibrotic disorders[5]. Systemic sclerosis (scleroderma, SSc) is a drug-orphan connective tissue disease falling into both of these categories, thus representing an ideal field of application of MSC-based treatments[6]. One of the most common clinical problems in SSc is pulmonary involvement, that causes interstitial lung abnormalities in about 90% of patients and interstitial lung disease (ILD) in 40–60% based on different studies [7]. Although many immunomodulatory drugs have been tried in the treatment of SSc-ILD, only cyclophosphamide (CYC) was found to be effective in stabilizing or improving lung function in randomized clinical trials[8, 9], but its beneficial effect seems to be short lived[10] and associated with significant morbidity and mortality[11]. Imatinib was tested in a prospective, phase II pilot study that included 30 SSc patients with active ILD, unresponsive to CYC, and resulted in an improvement or stabilization of lung disease in about 70% of patients[12]. Notwithstanding these possible treatments, alternative immunosuppressive therapy seems to be warranted[13], including haematopoietic stem cell transplantation[14] or other cell therapies. A variety of MSC have been isolated, characterized and tested experimentally in pre-clinical animal models and appear to be effective in limiting the initial inflammatory responses and in resolving fibrosis during lung injury. BM was the first source reported to contain MSC. However, for clinical use, BM may be detrimental due to the highly invasive donation procedure and to the decline in MSC number with increasing age. More recently, UC, attainable by a less invasive method, was introduced as an alternative source for MSC. UC-MSC have several properties that make them of interest for therapeutic use: i. they can be isolated in large numbers; ii. they have a stable surface marker expression in early passages (passages 4–8); iii. they grow robustly and can be frozen/thawed; iv. they can be clonally expanded; and v. they can be easily engineered to express exogenous proteins. Moreover, UC-MSC have been shown to be the MSC with the greatest immune suppressive properties, and this has been correlated to the high levels of HLA-G (non-classical HLA with strong immune-inhibitory properties) that these cells express, especially under IFNγ stimulation[15]. At the same time, human UC-MSC (hUC-MSC) express an immunosuppressive isoform of HLA-I, but not HLA-DR (major histocompatibility complex [MHC] class II cell surface receptor), which suggests that these cells have low immunogenicity[16]. Furthermore, the expression of immune response-related surface antigens, such as CD40, CD40 ligand, CD80, and CD86 is absent on hUC-MSC, facilitating hUC-MSC escape from the host immune attack[17]. Upon this evidence, we decided to evaluate the therapeutic potential of systemically administered hUC-MSC in the well established model of bleomycin-induced lung fibrosis. Unlike the only previous study reporting the efficacy of hUC-MSC in reducing bleomycin-induced lung injury, which was obtained in immunodeficient SCID mice[18], in the present study we aimed at demonstrating the anti-fibrotic activity of hUC-MSC in vivo in bleomycin-treated immunocompetent C57BL/6 mice, in order to make an experimental model representing an immunocompetent human subject affected by SSc-ILD.

## Materials and methods

### Ethics statement

The hUC-MSC were isolated from umbilical cord (UC) obtained from women undergoing cesarean section at the Obstetrics and Gynecology Unit, Papa Giovanni XXIII Hospital, Bergamo, Italy. The women undergoing cesarean section provided written informed consent to the use of their UC for research purposes. This procedure was approved by the ethics committee of the Hospital (authorization n. 1239/2017) (S1 File) and by Agenzia Italiana del Farmaco (AIFA), i.e. the Italian Medicines Agency (authorization aM—62/2014). Review and approval by AIFA included ethical approval of all research procedures involving human subjects. UC used in this study was collected by the obstetricians at the Obstetrics and Gynecology Unit, Papa Giovanni XXIII Hospital, Bergamo, Italy, between July 17^th^ and 24^th^, 2017. Within 24 hours upon collection, Dr. Chiara Capelli took the UC and started the isolation procedure of hUC-MSC as described below. All UC were fully anonymized prior to being accessed by Dr. Chiara Capelli.

All animal care and experimental procedures were approved by the Italian Ministry of Health (authorization n. 456/2016-PR) and performed according to the Declaration of Helsinki conventions.

### Isolation and characterization of hUC-MSC

The hUC-MSC were isolated from UC as previously described[19]. In detail, fresh human UC was collected from nine donors. Cords from mothers or newborns with questionable health status or HBV, HCV or HIV positive mothers were excluded. UC (20–30 cm) was collected in a transfer medium consisting of Phosphate Buffered Saline (PBS) without Ca2+ and Mg2+ enriched with 50 μg/ml Gentamicin and 25 IU/ml Heparin. UC was maintained at 4°C and processed within 24 hours upon collection. UC was transferred under a sterile laminar flow cell culture hood into a 150 mm Petri dish and washed 3 times with the same PBS medium; umbilical vein and arteries were rinsed with PBS to remove any trace of contaminant red blood cells.

UC was then cut into 5 cm long segments which were subsequently longitudinally cut and split open to expose the inner surface. The obtained segments were subsequently transferred to a new 150 mm Petri dish and minced in very small fragments using sterile scalpels.

Using a sterile disposable tweezer, the fragments obtained from the cutting of each UC segment of about 5 cm length were then transferred into one 150 mm cell culture Petri dish with 40 ml of MSC expansion medium consisting of alpha-MEM enriched with 5% human platelet lysate obtained from healthy donors, 50 μg/ml gentamicin and 2 UI/ml Heparin and incubated at 37°C in humidified atmosphere with 5% CO2. The dishes were left undisturbed for 6–7 days, then UC tissue was removed, the Petri dishes washed twice with sterile PBS and adherent cells were expanded in MSC expansion medium for one additional week. 40% of the medium was changed every 3–4 days. After approximately 14 days, adherent cells were harvested by TrypLe Select 1X treatment (passage 1, P1). Cells were then suspended in MSC expansion medium, counted by Trypan Blue dye and plated in multilayered flasks for two consecutive expansion steps (P2 and P3, respectively) at a density of 100–500 cells/cm^2^ until they reached near confluence (70–80%). Final product was then detached as before, washed in sterile saline + 1% Human Serum Albumin solution, immunophenotyped by flow cytometry (BD FACSCanto II, Beckton Dickinson) and frozen in 10% DMSO and 90% human AB plasma. The whole process from initial UC fragmentation to MSC expansion and insertion of final product in the cryopreservation bags and sealing was performed in a class A environment within a dedicated class B laboratory.

We used for this study only sterile, unsorted, viable cells characterized by: CD73, CD90 and CD105 expression ≥90%; CD14, CD34 and CD45 expression ≤5%; lack of colony forming capacity and chromosome aberrations. Prior to infusion into mice, hUC-MSC were thawed from cryopreservation bags under sterile hood, tested again to confirm their immunophenotype and viability, and assayed for human TSG-6 expression as described below.

### Mice

Twelve- to sixteen-week old female C57BL/6 mice (Charles River Laboratories) were used in all experiments. Mice were housed under pathogen-free conditions at Servizio di Allevamento e Sperimentazione Animale I.N.R.C.A., Ancona, Italy, and maintained as described[20]. All animal care and experimental procedures were approved by the Italian Ministry of Health (authorization n. 456/2016-PR) and performed according to the Declaration of Helsinki conventions. In detail, mice were maintained on a 12-hours light/dark cycle and were given free access to water and standard mouse chow. Any possible efforts were made to minimize animal suffering. All surgical procedures were performed under anesthesia, placing the mice on heated mats to keep the rectal temperature stable at 37°C throughout the intervention. In particular, buprenorphine (0.01 mg/kg diluted in sterile water) was administered subcutaneously to all mice before any surgical intervention and every 12 hours afterwards for two days in order to ensure prolonged analgesia and avoid any residual post-interventional pain. Moreover, after any invasive procedures mice were observed twice daily to monitor their health status and detect early any suffering or pathological signs.

### Bleomycin-induced lung injury mouse model and infusion with hUC-MSC

Mice were anaesthetized intraperitoneally with tribromoethanol (Avertin, Sigma-Aldrich) and lung injury was induced by a single endotracheal injection of 1.5 U/kg body weight of bleomycin sulfate (Sigma-Aldrich) in 100 μl of sterile saline. Control animals received endotracheal injection of equal volume of saline. Intravenous injection of hUC-MSC (2.5 × 10^5^ in 200 μl of sterile saline) into the tail vein was performed 24 hours and 7 days after bleomycin administration. This double infusion protocol was adapted from a previously published study [21]. Control animals received equal volume of sterile saline or equal number of primary human dermal fibroblasts isolated from adult skin (HDFa, Cat. no. C-013-5C, Invitrogen) into the tail vein. Successful intravenous (i.v.) infusion was monitored by lack of extravasation. Mice were sacrificed at days 8, 14 and 21 after bleomycin administration by isoflurane inhalation. Trachea and lungs were excised and immediately washed in ice-cold PBS. Right lungs were snap frozen in liquid nitrogen and stored at -80°C for molecular analysis. Left lungs were inflated with 4% paraformaldehyde and fixed in 10% Neutral Buffered Formalin Solution (Sigma-Aldrich) for 24 hours, then dehydrated in graded alcohol series, cleared in xylene and embedded in paraffin.

### Histology and immunohistochemistry

Paraffin-embedded lung tissue blocks were serially cut into 5 μm thick sections and processed as described[22]. Serial lung sections were stained with hematoxylin and eosin (H&E) (Bio-Optica, Milano, Italy) and Picrosirius Red. For immunohistochemistry, antigen retrieval was performed with a pressure cooker (90°C, 20 minutes), soaking the sections in 0.01 M sodium citrate buffer pH 6.0. After a rinse in PBS, sections were treated with 3% H_2_O_2_ in H_2_O at room temperature for 10 minutes to block endogenous peroxidase activity, rinsed with PBS and incubated with 5% Normal Serum (Vector Laboratories) in a humidified chamber at room temperature for 25 minutes. Mouse macrophages were stained with Rat Anti-Mouse Galectin-3 Monoclonal Antibody (1:1500; CL8942AP, Cedarlane Laboratories, Burlington, Ontario, Canada) or Mouse Anti-Mouse Arginase I Monoclonal Antibody (1:200, sc-271430, Santa Cruz Biotechnology). hUC-MSC were stained with Rabbit Anti-Human CD105 Monoclonal Antibody (1:50; Abcam) or Mouse Anti-Human HLA Class 1 ABC Monoclonal Antibody (1:200; Abcam), in a humidified chamber at 4°C overnight. After PBS rinse, sections were incubated with Biotinylated Rabbit Anti-Rat IgG Antibody, or Biotinylated Horse Anti-Mouse IgG Antibody, or Biotinylated Goat Anti-Rabbit IgG Antibody, or Biotinylated Horse Anti-Mouse IgG Antibody (all diluted 1:200; Vector Laboratories) in a humidified chamber at room temperature for 30 minutes. Immunoreactivity was developed using VECTASTAIN ABC Peroxidase Kit (Vector Laboratories) and SIGMA FAST 3,3′-Diaminobenzidine Tablets (Sigma-Aldrich). Sections were counterstained with haematoxylin, dehydrated and mounted with Eukitt Quick-Hardening Mounting Medium (Sigma-Aldrich). Immunohistochemistry for mouse alpha smooth muscle actin (α-SMA) was performed alike, using Rabbit anti-Mouse α-SMA polyclonal antibody (ab5694, Abcam; 1:150) followed by Biotinylated Goat Anti-Rabbit IgG. Immunohistochemistry for human vimentin was performed using the Dako Omnis Autostainer (Agilent, Santa Clara, CA) and the reagents of the EnVision FLEX, High pH (link) visualization system (Agilent, Santa Clara, CA) which include Peroxidase-Blocking Reagent, EnVision/HRP, Diaminobenzidine Chromogen, Substrate Buffer, Target Retrieval Solution, High pH (50x Tris/EDTA buffer, pH 9), and Wash Buffer (20x). Samples were deparaffinized and rehydrated prior to antigen retrieval at 95 °C for 30 minutes. Sections were then incubated for 20 minutes with the primary antibody, a rReady-to-use monoclonal mouse antibody provided in liquid form in a buffer containing stabilizing protein and 0.015 mol/L NaN_3_. Ready-to-use monoclonal mouse antibody provided in liquid form in a buffer containing stabilizing protein and 0.015 mol/L NaN_3_.

Clone: V9 (3). Isotype: Ig1, kapprready-to-use monoclonal mouse antibody (anti-vimentin, clone V9, Dako Omnis, Agilent, Santa Clara, CA), or the negative control agent (a non-specific isotype-matched primary antibody), and then with the Envision FLEX HRP for 20 minutes. The enzymatic conversion of the subsequently added diaminobenzidine chromogen resulted in precipitation of a visible reaction product localized to the antigen. Slides were counterstained with haematoxylin for 7 minutes, dehydrated and coverslipped. Staining was visualized using a light microscope and reaction specificity was ascertained by the absence of staining in sections incubated with the negative controls.

Lung sections were observed under a Nikon Eclipse E800 light microscope and digital images were captured with a Nikon DXM 1220 camera. Immunoreactive cells were counted in five randomly chosen high-power fields per section with Nikon LUCIA IMAGE software (version 4.61; Laboratory Imaging, Praha, Czech Republic). Cell count was performed by two independent observers in blind fashion. The average positive stained cell number per sample was used for statistical analysis.

### Histological scoring of lung inflammation

For the quantitative analysis of inflammatory changes in mouse lungs, hematoxylin-eosin-stained lung sections were observed under a light microscope and a histopathological inflammatory scoring system based on the inflammatory infiltration around bronchioles, bronchi, blood vessels and interstitial pneumonia was used[23]. Inflammatory changes in each lung sample were assessed as a mean score of severity from observed microscopic high-power fields per section and expressed on a numerical scale from 0 to 26 (least to most severe). Each lung specimen was scored by two independent observers in blind fashion.

### Histological scoring of lung fibrosis

For the quantitative analysis of fibrotic changes in mouse lungs, Picrosirius Red-stained lung sections were observed under a light microscope and a histopathological fibrotic scoring system based on alveolar and bronchiolar wall thickening and lung architecture damage was used[24]. Fibrotic changes in each lung sample were assessed as a mean score of severity from observed microscopic high-power fields per section and expressed on a numerical scale from 0 (normal lung) to 8 (most severe fibrosis). Each lung specimen was scored by two independent observers in blind fashion.

### Hydroxyproline assay

To quantify the amount of collagen in mouse lung specimens, hydroxyproline content was measured by Hydroxyproline Assay Kit (Sigma-Aldrich, #MAK008), according to the manufacturer’s instructions. Absorbance at 560 nm was determined with a microplate reader (Elisa Plate Reader, Tecan) and values were converted to hydroxyproline content using a standard curve. The collagen amount (μg) was expressed relatively to sterile saline-treated control mice.

### Determination of collagen and cytokines gene expression in mouse lungs by real-time quantitative PCR

Lung samples were homogenized in TRI Reagent (Sigma-Aldrich) and total RNA was extracted according to manufacturer’s instructions. RNA was further processed as described[25]. Specific primers for mouse genes were: Col1A1 forward, 5’-ACATGTTCACGTTTGTGGACC-3’; Col1A1 reverse: 5’-TAGGCCATTGTGTATGCAGC-3’. IL-1 forward, 5’-CCCAAGCAA TACCCAAAGAA-3’; IL-1 reverse, 5’-CATCAGAGGCAAGGAGGAAA-3’; IL-2 forward, 5’-ATGTACAGCATCCAGCTCGCATC-3’; IL-2 reverse, 5’-GGCTTGTTGAGATG ATGCTTTGACA-3’; IL-6 forward, 5’-TTCCATCCAGTTGCCTTCTT-3’; IL-6 reverse, 5’-ATTTCCACGATTTCCCAGAG-3’; IL-10 forward, 5’-TTGAGTCTGCTGGACT CCAGGACCTAGACA-3’; IL-10 reverse, 5’-GCAGCCAAACAATACACCATTCCCAGA GG-3’; TGFβ forward, 5’-CTACTGCTTCAGCTCCACAG-3’; TGFβ reverse, 5’-GCA CTTGCAGGAGCGCAC-3’; α-SMA forward, 5’-GTTCAGTGGTGCCTCTGTCA-3’; α-SMA reverse, 5’-ACTGGGACGACATGGAAAAG-3’; Cyclophilin forward, 5’-GTGTTCTTCGACATCACGGC-3’; Cyclophilin reverse, 5’-GTGTT CTTCGACATCACGGC-3’; 18S rRNA forward, 5’-AGTCCCTGCCCTTTGTACACA-3’; 18S rRNA reverse, 5’-CGATCCGAGGGCCTCACTA-3’; Human/Mouse GAPDH forward, 5’-CAGCGACACCCACTCCTCCAC CTT-3’; Human/Mouse GAPDH reverse, 5’-CATGAGGTCCACCACCCTG TTGCT-3’.

Reactions were performed in triplicate for each sample, in a volume of 25 μl containing 50 nanograms of cDNA, 12,5 μl iQ SYBR Green Supermix (Bio-Rad) and 400 nM of primers. Cycling parameters were: denaturation at 95°C for 15 seconds and annealing at 60°C for 1 minute, for 40 cycles. After normalizing data to GAPDH, cyclophilin and 18S rRNA, gene expression relative to sterile saline-treated control mice was calculated by the ΔΔCt method.

### Protein isolation and Western blotting

Snap-frozen lungs were placed in an appropriate volume (10 mg tissue/100 μl lysis buffer) of RIPA lysis buffer supplemented with Protease and Phosphatase Inhibitors Cocktail (Cell Signaling Technology) and mechanically homogenized using gentleMACS Dissociator (Miltenyi Biotec). Protein concentration of lung homogenates was measured by Bradford Assay (Bio-Rad). 50 μg of total proteins were loaded onto 4–12% Bolt Bis-Tris Plus polyacrylamide gels (ThermoFisher Scientific), transferred onto nitrocellulose membrane and blocked for 2 hours in TBS + 0,5% Tween-20 (TBS-T) containing 5% Blotto Non-Fat Dry Milk (Santa Cruz Biotechnology). Rabbit anti-IL-1β (Abcam, #ab9722, 1:1500), mouse anti-IL-2 (F-5) (Santa Cruz Biotechnology, #sc-133118, 1:500), mouse anti-IL-6 (10E5) (Santa Cruz Biotechnology, #sc-57315, 1:500), mouse anti-IL-10 (A-2) (Santa Cruz Biotechnology, #sc-365858, 1:500), mouse anti-TGFβ1 (3C11) (Santa Cruz Biotechnology, #sc-130348, 1:500), mouse anti-COL1A1 (3G3) (Santa Cruz Biotechnology, #sc-293182, 1:500) primary antibodies were then added to membranes and incubated at 4 °C overnight. Blots were washed with TBS + 0.5% Tween-20 and secondary antibodies, including goat anti rabbit IgG-HRP (Santa Cruz Biotechnology, #sc-2004) and goat anti-mouse IgG-HRP (Santa Cruz Biotechnology, #sc-2005) were added for 1 hour. Blots were finally imaged using ChemiDoc^™^ Imaging System (Bio-Rad). Quantification of protein signals was performed by densitometry using Quantity One Basic software (Bio-Rad).

### Detection of hUC-MSC in mouse lungs

Detection of hUC-MSC in mouse lungs was performed not only by immunohistochemistry, as described above, but also by real-time PCR with human-specific GAPDH primers and probe (TaqMan Gene Expression Assays ID, Hs00266705_g1). Standard curves were generated by serial dilutions of cDNA obtained from cultured hUC-MSC prior to infusion.

### Analysis of TSG-6 expression in hUC-MSC and mouse lungs

Human TSG-6 expression was measured by quantitative real-time PCR. Specific primers and probe were: forward, 5’-AAGCACGGTCTGGCAAATACAAGC-3’; reverse, 5’-ATCCATCCAGCAGCACAGACATGA- 3’; probe, 5’-TTTGAAGGCGGCCATCT CGCAACTT-3’. Reactions were performed, in triplicate for each sample, in a volume of 25 μl containing 50 ng target template, 12.5 μl iQ Supermix (Bio-Rad), 400 nM primers and 250 nM probe. Cycling parameters were: denaturation at 95°C for 30 seconds and annealing at 60°C for 1 minute, for 40 cycles. Standard curves were generated by serial dilutions of cDNA obtained from cultured hUC-MSC prior to infusion. Final data were normalized by parallel real-time PCR with primers that amplified both the human and the mouse genes for GAPDH (listed above).

### Statistical analysis

All quantitative data were expressed as mean ± SD. Data were analyzed using Mann-Whitney test for nonparametric values, unpaired and two-tailed Student t test for parametric values and one-way ANOVA to compare more than two groups. All statistical analyses were performed using GraphPad Prism 5 software (California, USA). P values < 0.05 were considered significant.

## Results

### hUC-MSC down-regulate bleomycin-induced lung inflammation and fibrosis

**Fig 1:**
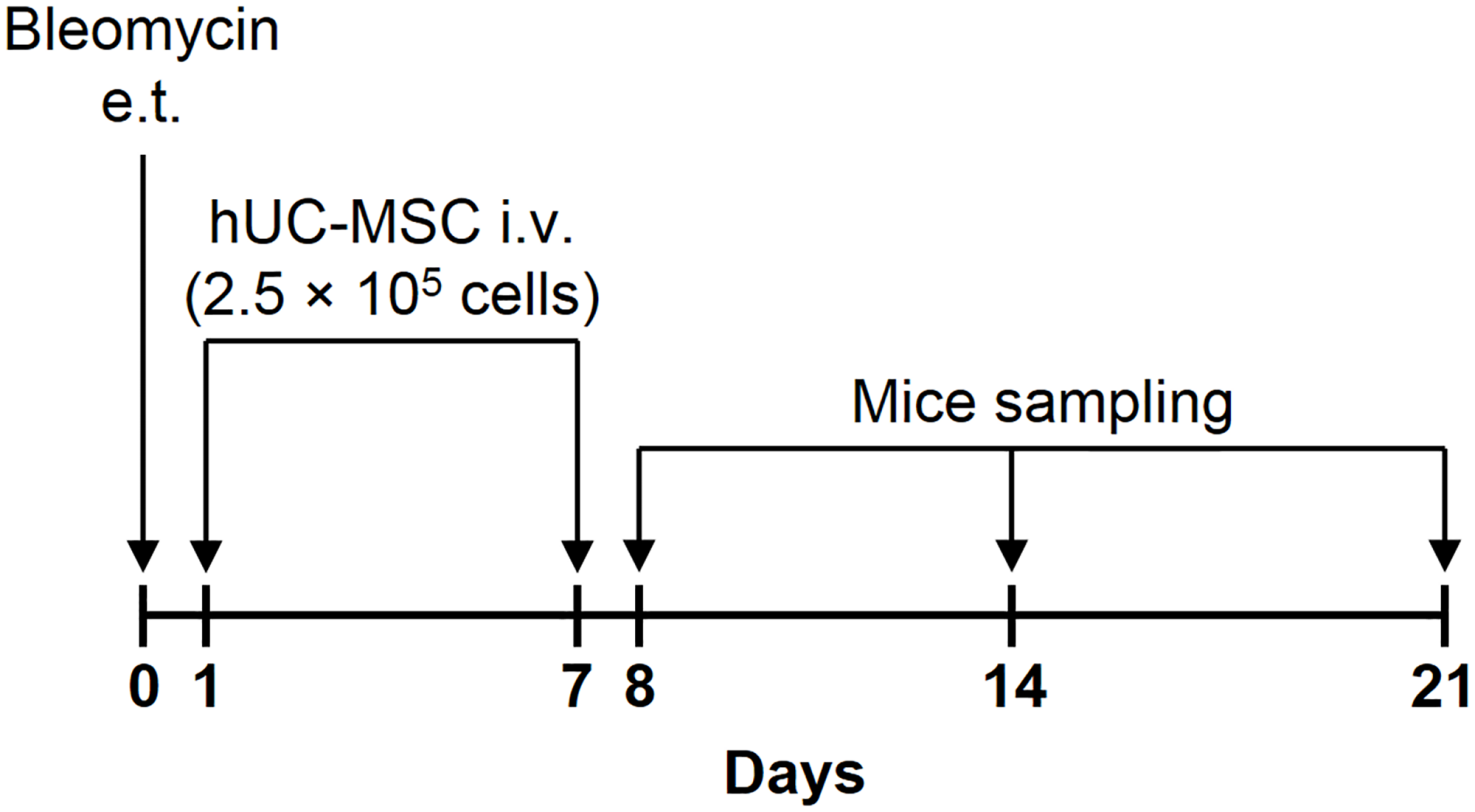
Schematic of the experimental model. Mice received a single endotracheal (e.t.) injection of 1.5 U/kg body weight of bleomycin sulfate to induce lung injury (day 0). Intravenous (i.v.) injection of human mesenchymal stromal cells obtained from whole umbilical cord (hUC-MSC) (2.5 × 10^5^ cells) into the tail vein was performed 24 hours (day 1) and 7 days (day 7) after bleomycin administration. Mice groups were sacrificed at days 8, 14 and 21 after bleomycin administration (i.e., 24 hours, 7 days and 14 days after second hUC-MSC infusion, respectively).

**Fig 2:**
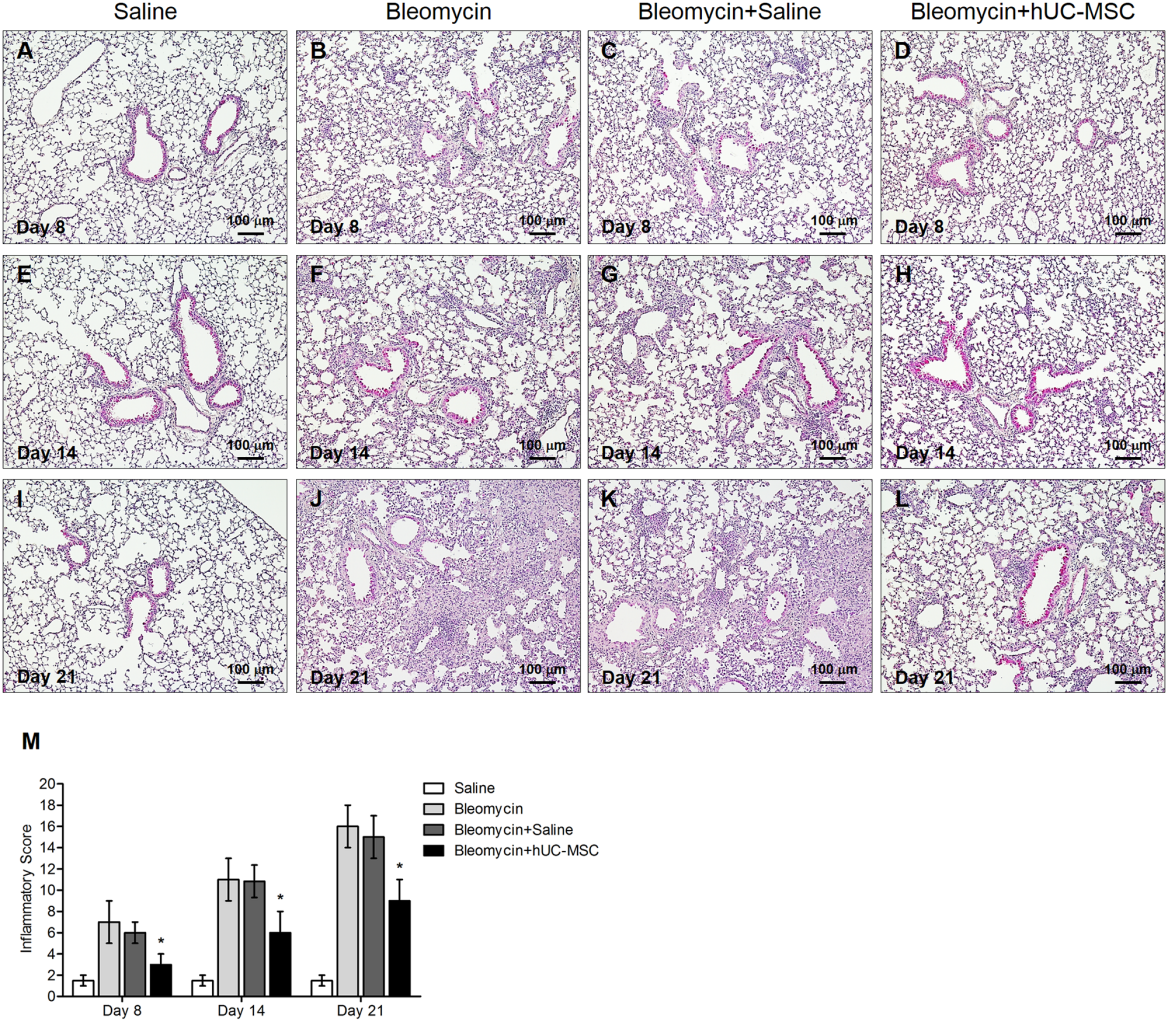
hUC-MSC down-regulate bleomycin-induced lung inflammation. Histology of mouse lungs 8 days (**A-D**), 14 days (**E-H**) and 21 days (**I-L**) after endotracheal injection of sterile saline (saline) or bleomycin (bleomycin), the latter also followed by intravenous infusion of hUC-MSC (bleomycin+hUC-MSC) or sterile saline (bleomycin+saline). Lung sections obtained from C57BL/6 mice (n = 8 per group) were stained with H&E. Controls (**A,E,I**) demonstrated normal lung architecture. 8 days post bleomycin injury, peribronchial and perivascular inflammatory infiltrates were observed (**B**). Alveolar and interstitial infiltrates progressively increased from day 8 to 21, with progressive distortion of lung architecture and formation of fibrotic foci (**F,J**). At each time point, bleomycin-induced alterations were significantly attenuated by hUC-MSC treatment (**D,H,L**), but not by saline (**C,G,K**). Representative microscopic images (10× magnification) of three independent experiments are shown. (**M**) The histopathological inflammatory score of lung sections obtained from C57BL/6 mice (n = 8 per group) that received the aforementioned treatments was calculated. Results are mean ± SD (n = 8 per group) and are representative of three independent experiments. * = P < 0.05 compared to bleomycin treated mice.

**Fig 3:**
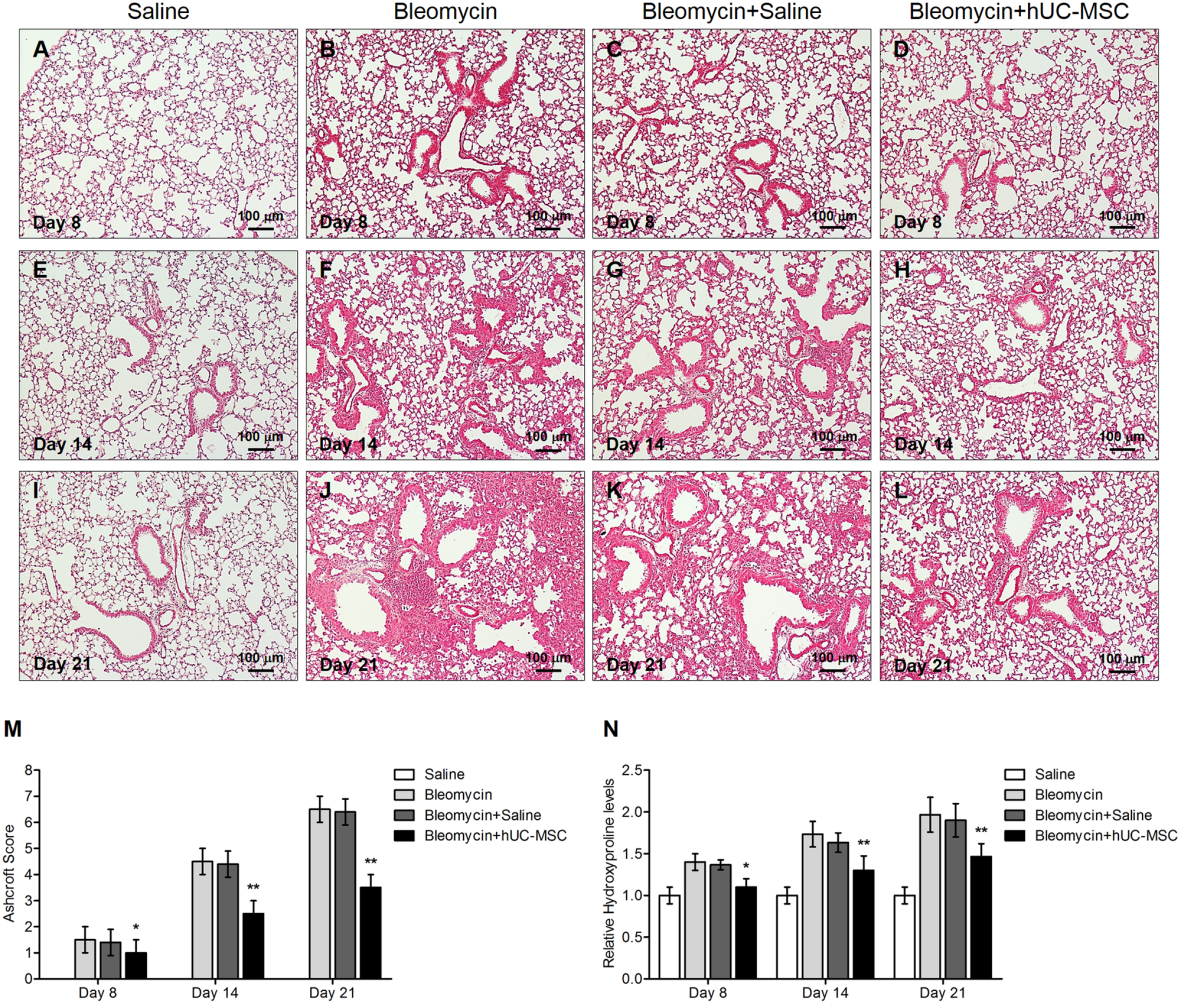
hUC-MSC down-regulate bleomycin-induced lung fibrosis. Collagen content in mouse lungs 8 days (**A-D**), 14 days (**E-H**) and 21 days (**I-L**) after endotracheal injection of sterile saline (saline) or bleomycin (bleomycin), the latter also followed by intravenous infusion of hUC-MSC (bleomycin+hUC-MSC) or sterile saline (bleomycin+saline). Lung sections obtained from C57BL/6 mice (n = 8 per group) were stained with Picrosirius Red. Controls (**A,E,I**) demonstrated normal lung architecture. 8 days post bleomycin injury, initial thickening of the alveoli and septa was observed (**B**). Collagen deposition progressively increased from day 8 to 21, with progressive distortion of lung architecture and formation of fibrotic foci (**F,J**). At each time point, bleomycin-induced alterations were significantly attenuated by hUC-MSC treatment (**D,H,L**), but not by saline (**C,G,K**). Representative microscopic images (10× magnification) of three independent experiments are shown. (**M**) The Ashcroft fibrosis score of lung sections obtained from C57BL/6 mice (n = 8 per group) that received the aforementioned treatments was calculated. Results are mean ± SD (n = 8 per group) and are representative of three independent experiments. * = P < 0.05, ** = P < 0.01, compared to bleomycin treated mice. (**N**) Hydroxyproline content in C57BL/6 mouse lungs that received the aforementioned treatments. Results are mean ± SD (n = 8 per group) expressed as a percent of the value obtained from endotracheal saline treated mice and are representative of three independent experiments. * = P < 0.05, ** = P < 0.01 compared to bleomycin treated mice.

Histological analysis of mouse lung tissue sampled at days 8, 14 and 21 following a single endotracheal injection of bleomycin (1.5 U/kg body weight) into C57BL/6 mice (n. = 8) (Fig 1) documented a prominent lung inflammation, with a diffuse alveolar and interstitial inflammatory cell infiltration in both lungs (Fig 2B, 2F and 2J), accompanied by massive fibrosis, as shown by collapsed and thickened alveoli, perivascular collagen deposition, bronchoalveolar hyperplasia and distortion of the normal architecture of the lung (Fig 3B, 3F and 3J). Sterile saline-treated control mice (n. = 8) demonstrated normal lung histology (Figs 2A, 2E, 2I, 3A, 3E and 3I). Bleomycin-induced pneumonia was largely attenuated in an equal number of mice by a double infusion of hUC-MSC (2.5 × 10^5^) into the tail vein, the first one 24 hours and the second one 7 days post-injury (Fig 1), with significant reduction of both the inflammatory infiltration evaluated by H&E staining (Fig 2D, 2H and 2L) and the extent of fibrosis evaluated by Picrosirius Red staining (Fig 3D, 3H and 3L). Conversely, a double i.v. infusion of saline solution did not block the pro-inflammatory, pro-fibrotic effect of bleomycin (Figs 2C, 2G, 2K, 3C, 3G and 3K). Inflammatory and Ashcroft scores confirmed the progression between days 8 and 21 of the lung inflammatory/fibrotic process induced by bleomycin, and its significant attenuation by hUC-MSC at each time point (Figs 2M and 3M). To better assess fibrosis with and without hUC-MSC therapy, quantification of total soluble and insoluble lung collagen content was performed using the gold standard hydroxyproline assay [26], which confirmed a statistically significant decrease in collagen content in the lungs of hUC-MSC -treated mice, especially at day 14 and 21 after bleomycin administration (Fig 3M).

**Fig 4:**
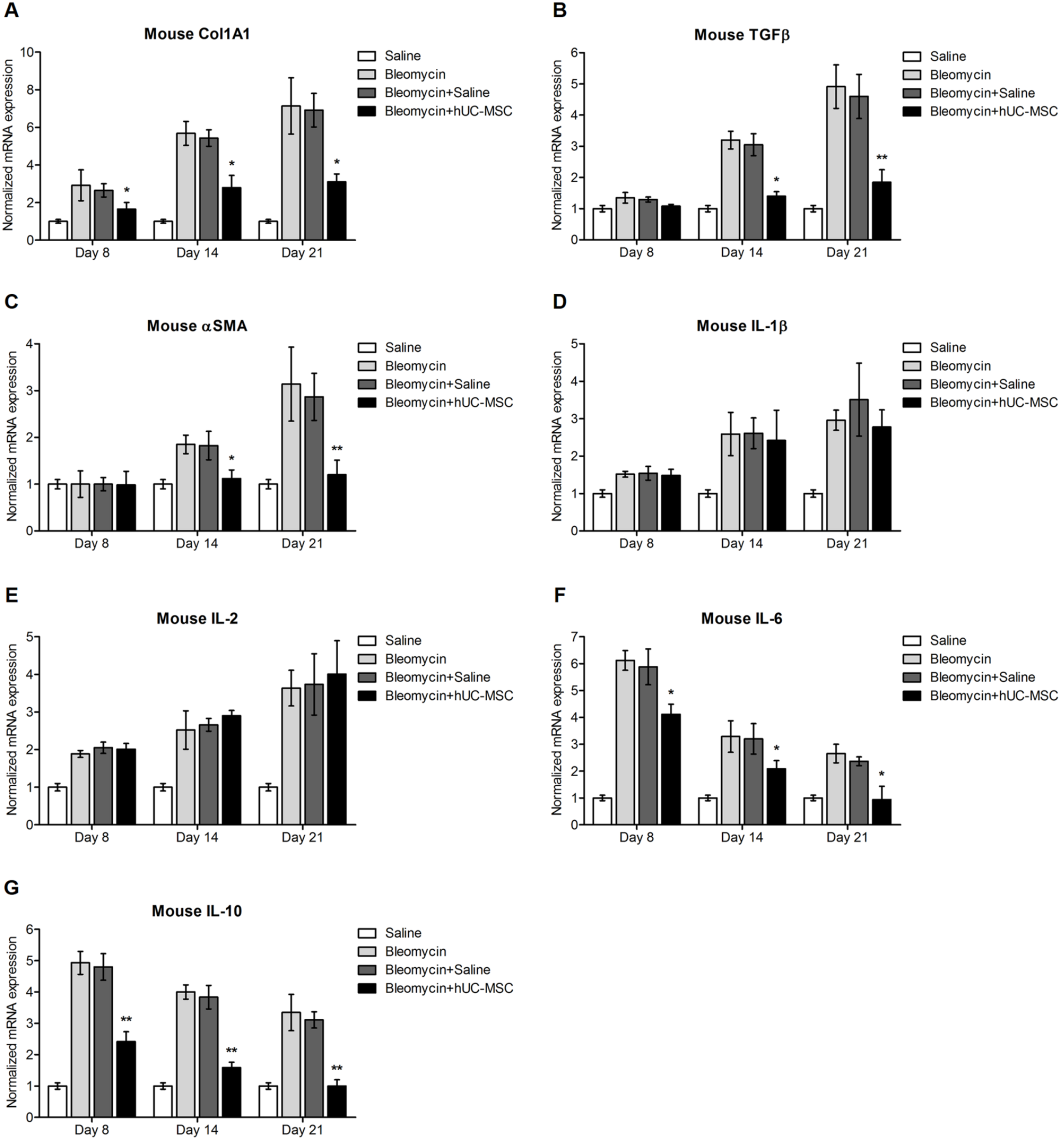
Time course of cytokines and matrix components in lung tissue assessed by quantitative real-time PCR. Gene expression analysis of Col1A1 (**A**), TGFβ (**B**), α-SMA (**C**), IL-1β (**D**), IL-2 (**E**), IL-6 (**F**) and IL-10 (**G**) in whole lung mRNA obtained at days 8, 14 and 21 after endotracheal injection of sterile saline (saline) or bleomycin (bleomycin), the latter also followed by intravenous infusion of hUC-MSC (bleomycin+hUC-MSC) or sterile saline (bleomycin+saline). Results are expressed as mean ± SD (n = 5 per group) and are representative of three independent experiments performed in triplicate. * = P < 0.05, ** = P < 0.01 compared to bleomycin treated mice.

**Fig 5:**
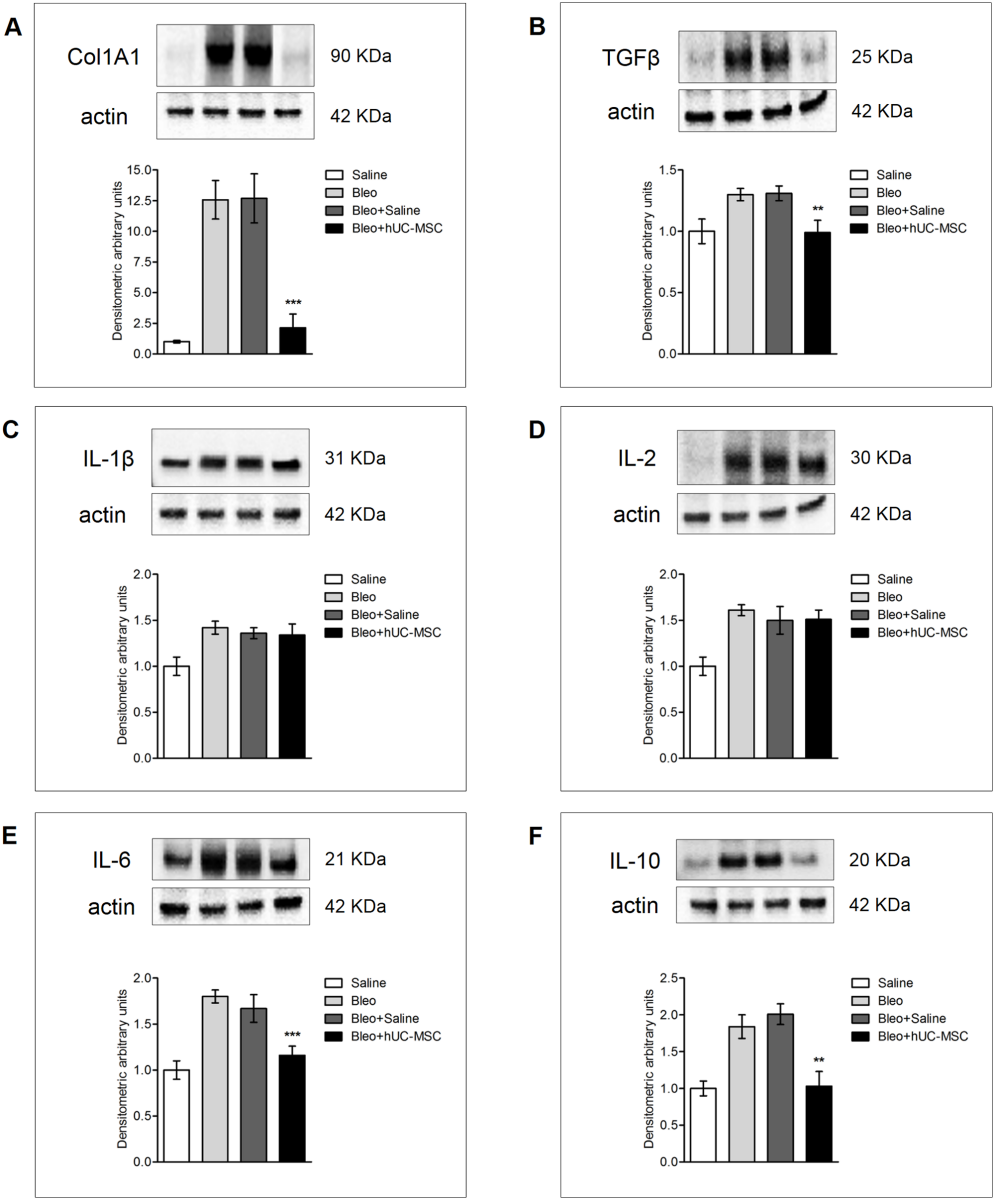
Protein expression of cytokines and matrix components in lung tissue assessed by Western blotting. Collagen Col1A1 (**A**), TGFβ (**B**), IL-1β (**C**), IL-2 (**D**), IL-6 (**E**) and IL-10 (**F**) protein levels in whole lung tissue lysates obtained from C57BL/6 mice 21 days after endotracheal injection of sterile saline (saline) or bleomycin (bleo), the latter also followed by intravenous infusion of hUC-MSC (bleo+hUC-MSC) or sterile saline (bleo+saline). Results are representative of three independent experiments. Densitometric analysis of protein bands is normalized to actin and expressed as mean ± SD (n = 5 per group). ** = P < 0.01, *** = P < 0.001 compared to bleomycin treated mice.

To determine if reduced collagen deposition was due to reduced collagen synthesis, Col1A1 gene expression was analyzed by quantitative real-time PCR in mRNA extracted from lungs. In hUC-MSC-treated mice we observed a significant decrease in Col1A1 mRNA expression at days 8, 14 and 21 post bleomycin-induced lung injury. Even if Col1A1 mRNA levels were still higher than in lungs taken from mice without bleomycin challenge, Col1A1 transcripts did not increase from day 14 to day 21, as opposed to the observed increase without hUC-MSC-treatment. In contrast to hUC-MSC, i.v. injection of sterile saline into bleomycin-injured mice did not reduce collagen gene expression (Fig 4A). Collagen gene expression data were confirmed by protein measurement using Western blotting (Fig 5A).

**Fig 6:**
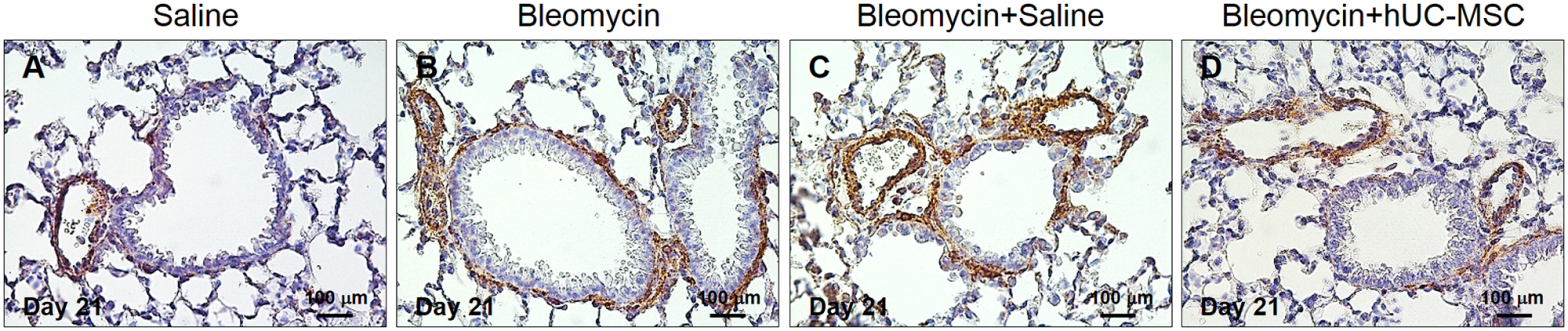
Immunohistochemical determination of α-SMA in lung tissue. Peribronchial and perivascular deposition of α-SMA in C57BL/6 mouse lungs at day 21 after injection of endotracheal sterile saline only (**A**), endotracheal bleomycin only (**B**), endotracheal bleomycin followed by intravenous sterile saline (**C**) or endotracheal bleomycin followed by intravenous hUC-MSC (**D**). Lung sections were immunostained with anti-α-SMA antibody. Representative microscopic images (40× magnification) of three independent experiments are shown.

Enhanced collagen deposition in bleomycin-treated mice was accompanied by increased expression of TGFβ, a cytokine known to mediate fibrosis, and αSMA, a marker of fibroblasts with collagen-secreting phenotype. Consistently, both transcripts were significantly down-regulated by hUC-MSC administration (Fig 4B and 4C). Importantly, mRNA data were confirmed by Western blotting (Fig 5B) and IHC (Fig 6), respectively. Thus, our results show that systemic administration of hUC-MSC has the ability to mitigate bleomycin-induced lung inflammation and fibrosis in C57BL/6 mice.

### The attenuation of bleomycin-induced fibrosis by hUC-MSC is associated with down-regulation of M2 macrophage activation

To investigate the mechanisms through which hUC-MSC may affect the immune response to bleomycin challenge and exert their anti-inflammatory, anti-fibrotic effect, mRNA was extracted from whole lung tissue obtained from hUC-MSC-treated and untreated mice at days 8, 14, and 21 after bleomycin instillation and transcripts were analyzed by quantitative real-time PCR. Lungs exposed to bleomycin displayed a significant increase in the expression of pro-inflammatory cytokines IL-1β, IL-2, IL-6 and anti-inflammatory cytokine IL-10, in comparison with sterile saline-treated control mice. Notably, following hUC-MSC treatment, the expression of pro-inflammatory cytokines IL-1 and IL-2 remained elevated whereas IL-6 and IL-10 were progressively reduced, down to the day 21 levels observed in saline-treated control mice (Fig 4D–4G). Cytokine gene expression data were confirmed by protein measurement at day 21 using Western blotting (Fig 5C–5F). Since IL-6 and IL-10 are secreted, albeit not exclusively, by M1 and M2 macrophages[27, 28], that have been implicated in tissue repair and subsequent organ fibrosis[29], we employed two different monoclonal antibodies, one targeting galectin-3, a general macrophage marker of chronic inflammation and fibrosis during bleomycin-induced fibrosis [30–32], the other one targeting a more specific marker of M2 macrophage activation such as arginase-1[33], to specifically label these cells in lungs taken from the experimental mice.

**Fig 7:**
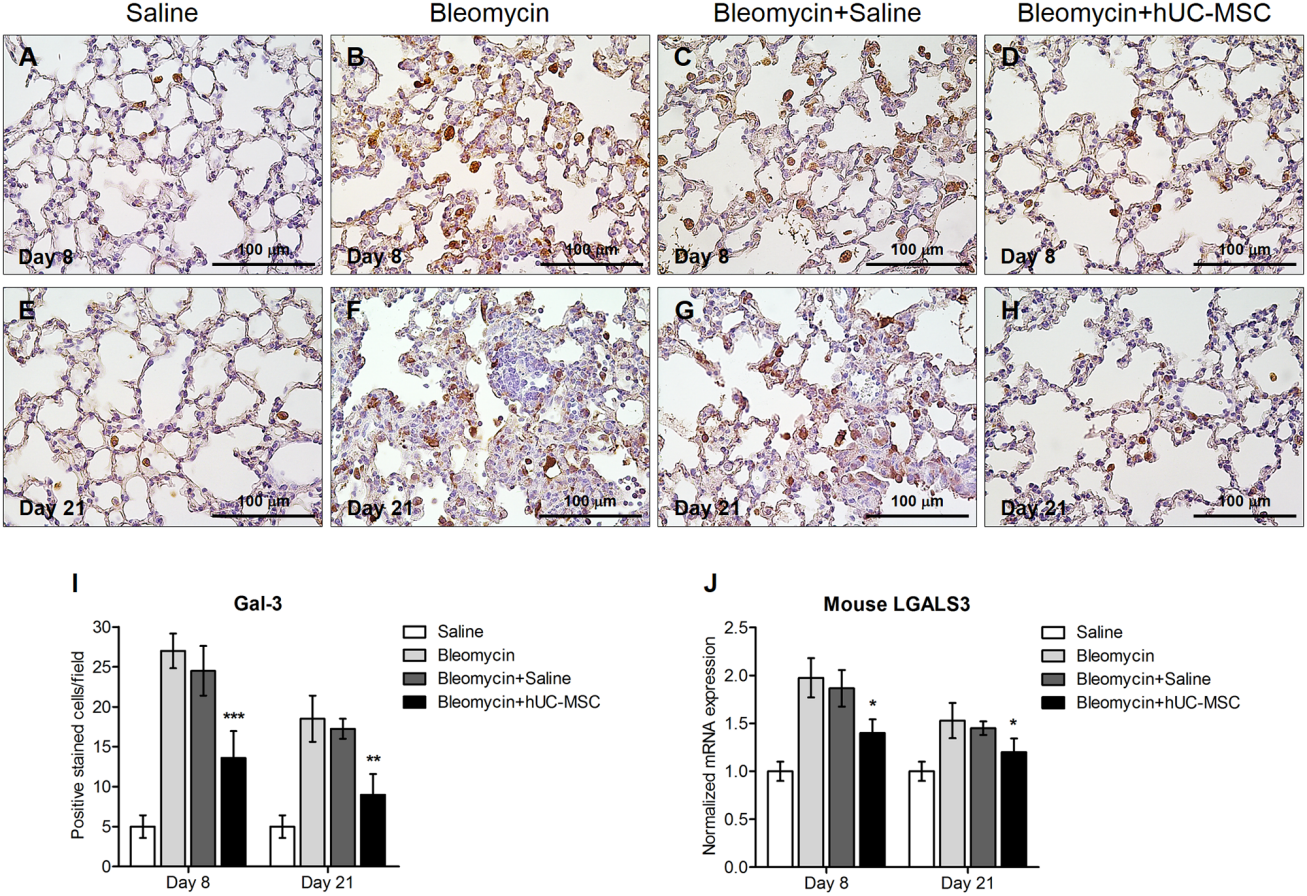
Decrease of galectin-3 positive cells in bleomycin-injured lung tissue upon administration of hUC-MSC. Macrophage infiltration in mouse lungs at days 8 (**A-D**) and 21 (**E-H**) after endotracheal injection of sterile saline (saline) (**A, E**) or bleomycin (bleomycin) (**B, F**), the latter also followed by intravenous infusion of hUC-MSC (bleomycin+hUC-MSC) (**D, H**) or sterile saline (bleomycin+saline) (**C, G**). Lung sections obtained from C57BL/6 mice (n = 8 per group) were immunostained with anti-galectin-3 antibody. At each time point, the number of immunoreactive macrophages infiltrating the lungs are significantly decreased by hUC-MSC infusion in comparison to bleomycin-treated mice. Representative microscopic images (40× magnification) of three independent experiments are shown. (**I)** Cell count of galectin-3 positive macrophages in C57BL/6 mouse lung sections, and (**J**) quantitative real-time PCR analysis of galectin-3 gene expression in whole lung mRNA, obtained at days 8 and 21. Results are expressed as mean ± SD of positively immunostained cell count per sample (n = 5 per group) and are representative of three independent experiments. * = P < 0.05, ** = P < 0.01, *** = P < 0.001 compared to bleomycin treated mice.

**Fig 8:**
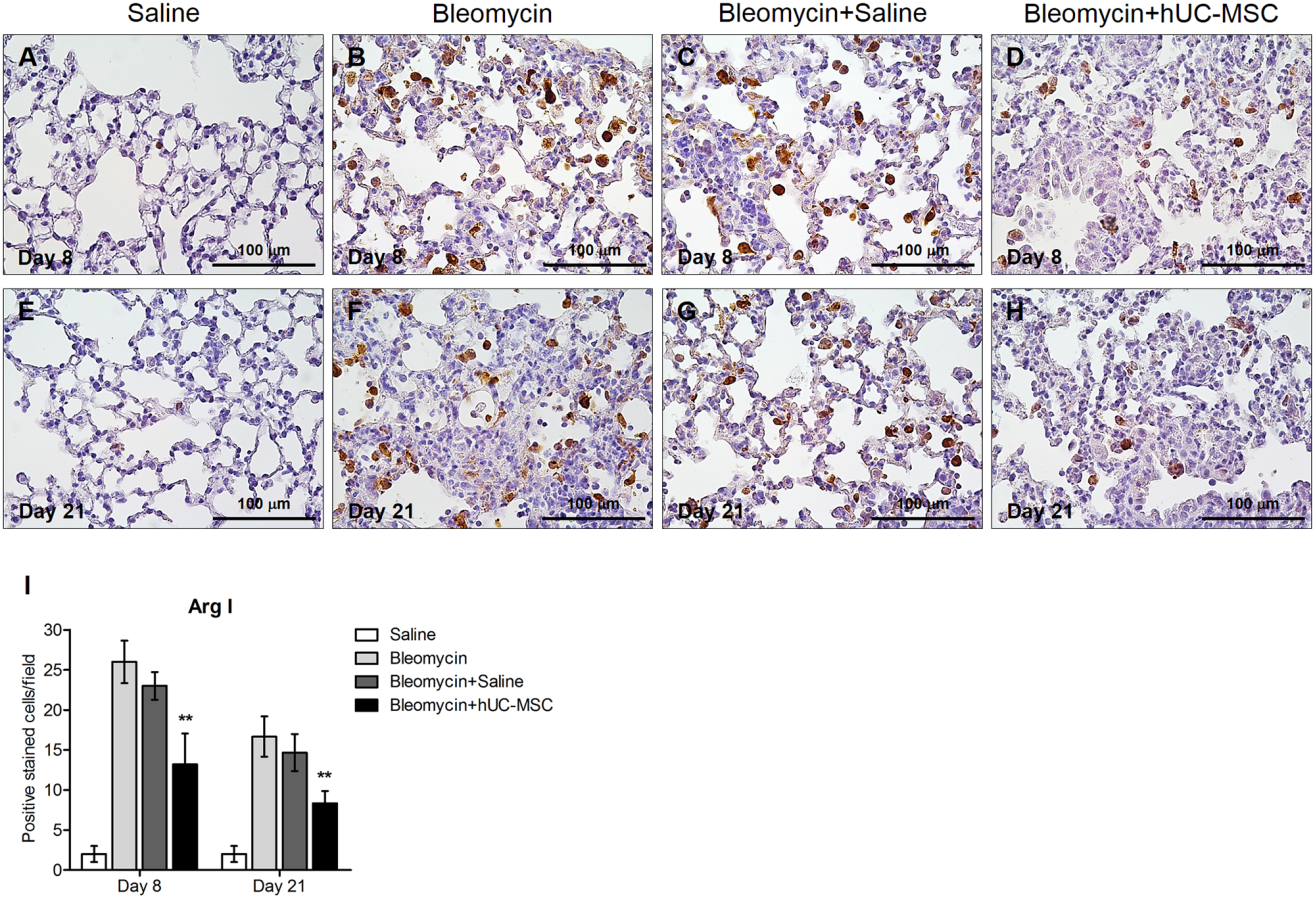
Decrease of arginase-I positive cells in bleomycin-injured lung tissue upon administration of hUC-MSC. Macrophage infiltration in mouse lungs at days 8 (**A-D**) and 21 (**E-H**) after endotracheal injection of sterile saline (saline) (**A, E**) or bleomycin (bleomycin) (**B, F**), the latter also followed by intravenous infusion of hUC-MSC (bleomycin+hUC-MSC) (**D, H**) or sterile saline (bleomycin+saline) (**C, G**). Lung sections obtained from C57BL/6 mice (n = 8 per group) were immunostained with anti-Arginase I antibody. At each time point, the number of immunoreactive macrophages infiltrating the lungs is significantly diminished by hUC-MSC infusion in comparison to bleomycin-treated mice. Representative microscopic images (40× magnification) of three independent experiments are shown. (**I)** Cell count of arginase I positive macrophages in C57BL/6 mouse lung sections. Results are mean ± SD of positive immunostained cell count per sample (n = 8 per group) and are representative of three independent experiments. ** = P < 0.01 compared to bleomycin treated mice.

We could observe a significant increase of Galectin-3 and arginase-1 positively stained macrophage cells count in the alveoli and interstitial spaces compared to saline-treated controls, which was significantly, although not completely, reverted by hUC-MSC administration both at day 8 and 21 post bleomycin-induced lung injury (Figs 7 and 8). Thus, our results show that systemic administration of hUC-MSC has the ability to specifically down-regulate the IL-6/IL-10/TGFβ axis involving lung macrophages, especially the M2 subset, the decrease of which correlates with the reduction of bleomycin-induced fibrosis in C57BL/6 mice.

### The down-regulation of bleomycin-induced injury and M2 macrophage activation is specific to hUC-MSC

To make sure that the down-regulation of bleomycin-induced injury observed with hUC-MSC was specific to this cell type, we treated additional bleomycin-injured mice with a double intravenous infusion of an equal number of a different mesenchymal cell type, that is primary human dermal fibroblasts isolated from adult skin.

**Fig 9:**
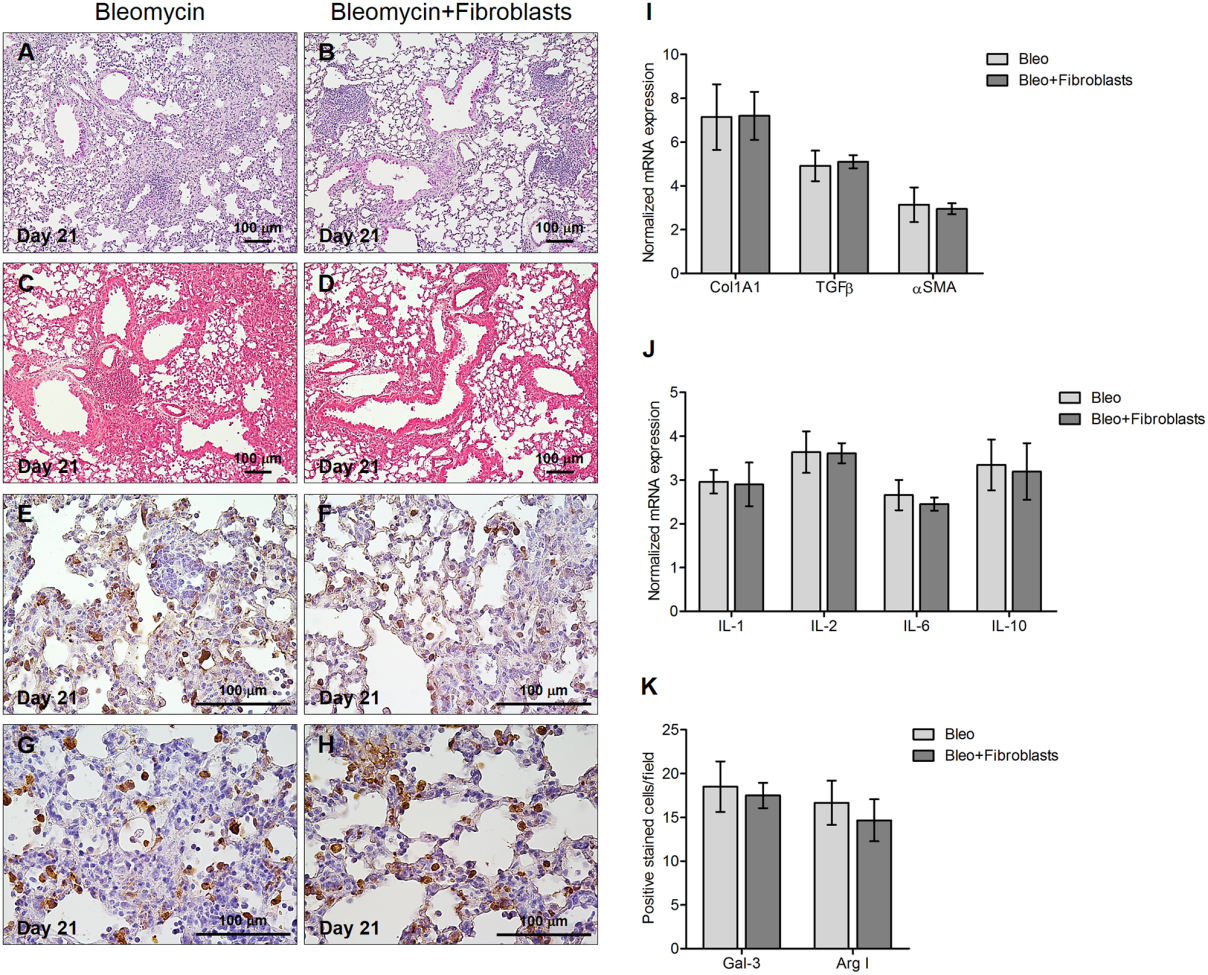
Intravenous administration of human fibroblasts does not prevent the development of bleomycin-induced lung injury. Histology (**A-B**), collagen content (**C-D**) and macrophage infiltration (**E-H**) of mouse lungs 21 days after bleomycin endotracheal injection followed by human fibroblasts intravenous infusion. Lung sections obtained from C57BL/6 mice (n = 8 per group) that received endotracheal bleomycin only (bleomycin) or endotracheal bleomycin followed by intravenous human fibroblasts (bleomycin+fibroblasts) were stained with H&E (**A-B**), Picrosirius Red (**C-D**), anti-galectin-3 (**E-F**) or anti-arginase I (**G-H**) antibodies. Representative microscopic images (10× or 40× magnification) of three independent experiments are shown. Quantitative real-time PCR gene expression analysis of Col1A1, TGFβ and αSMA (**I**), and IL-1β, IL-2, IL-6 and IL-10 (**J**) in whole lung mRNA obtained at day 21 from C57BL/6 mice receiving the aforementioned treatments. Results are expressed as mean ± SD (n = 5 per group) and are representative of three independent experiments performed in triplicate. (**K**) Quantification of galectin-3 and arginase-I positive macrophages in C57BL/6 mouse lungs 21 days after bleomycin injection with or without subsequent human fibroblast infusion. Results are mean ± SD of positive immunostained cell count per sample (n = 8 per group) and are representative of three independent experiments.

Bleomycin-induced lung alterations, both at histological and cellular and molecular levels, were not affected by this treatment (Fig 9), indicating that human fibroblasts do not share the anti-inflammatory, anti-fibrotic properties of hUC-MSC.

### hUC-MSC do not possess immunogenic activity in absence of bleomycin injury

**Fig 10:**
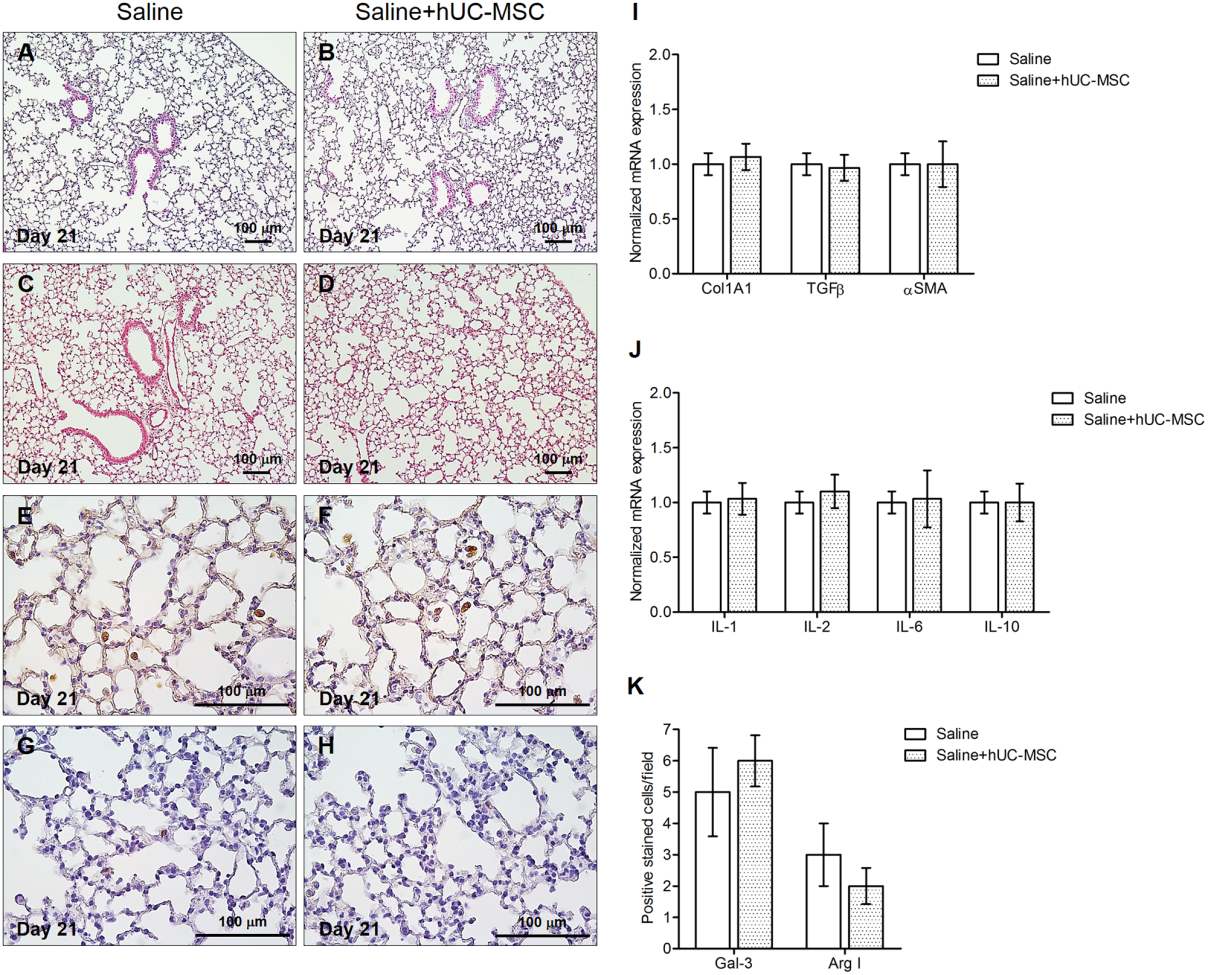
hUC-MSC do not induce any biological effects on control mice treated with endotracheal saline. Histology (**A-B**), collagen content (**C-D**) and macrophage infiltration (**E-H**) of mouse lungs 21 days after sterile saline endotracheal injection followed by hUC-MSC intravenous infusion. Lung sections obtained from C57BL/6 mice (n = 8 per group) that received endotracheal sterile saline only (saline) or endotracheal sterile saline followed by intravenous hUC-MSC (saline+hUC-MSC) were stained with H&E (**A-B**), Picrosirius Red (**C-D**), anti- galectin-3 (**E-F**) and anti-arginase I (**G-H**) antibodies. Representative microscopic images (10× or 40× magnification) of three independent experiments are shown. Quantitative real-time PCR gene expression analysis of Col1A1, TGFβ and αSMA (**I**), and IL-1β, IL-2, IL-6 and IL-10 (**J**) in whole lung mRNA obtained at day 21 from C57BL/6 mice receiving the aforementioned treatments. Results are expressed as mean ± SD (n = 5 per group) and are representative of three independent experiments performed in triplicate. (**K**) Quantification of galectin-3 and arginase I positive macrophages in C57BL/6 mouse lungs 21 days after saline injection with or without subsequent hUC-MSC infusion. Results are mean ± SD of positive immunostained cell count per sample (n = 8 per group) and are representative of three independent experiments.

Another set of control experiments was dedicated to assess if hUC-MSC may elicit changes in the expression of cytokine and matrix components, and in macrophage polarization, when systemically administered to mice injected with saline vehicle control only, but not with bleomycin. Remarkably, no significant changes were observed in the lungs of mice, either at histological or cellular or molecular levels (Fig 10). Consistently, no clinically relevant side effects such as fever, joint swelling, skin rash, or any other suffering manifestations were observed during the accurate surveillance of mice after both hUC-MSC i.v. administrations.

### Kinetics of hUC-MSC in lungs after systemic administration

**Fig 11:**
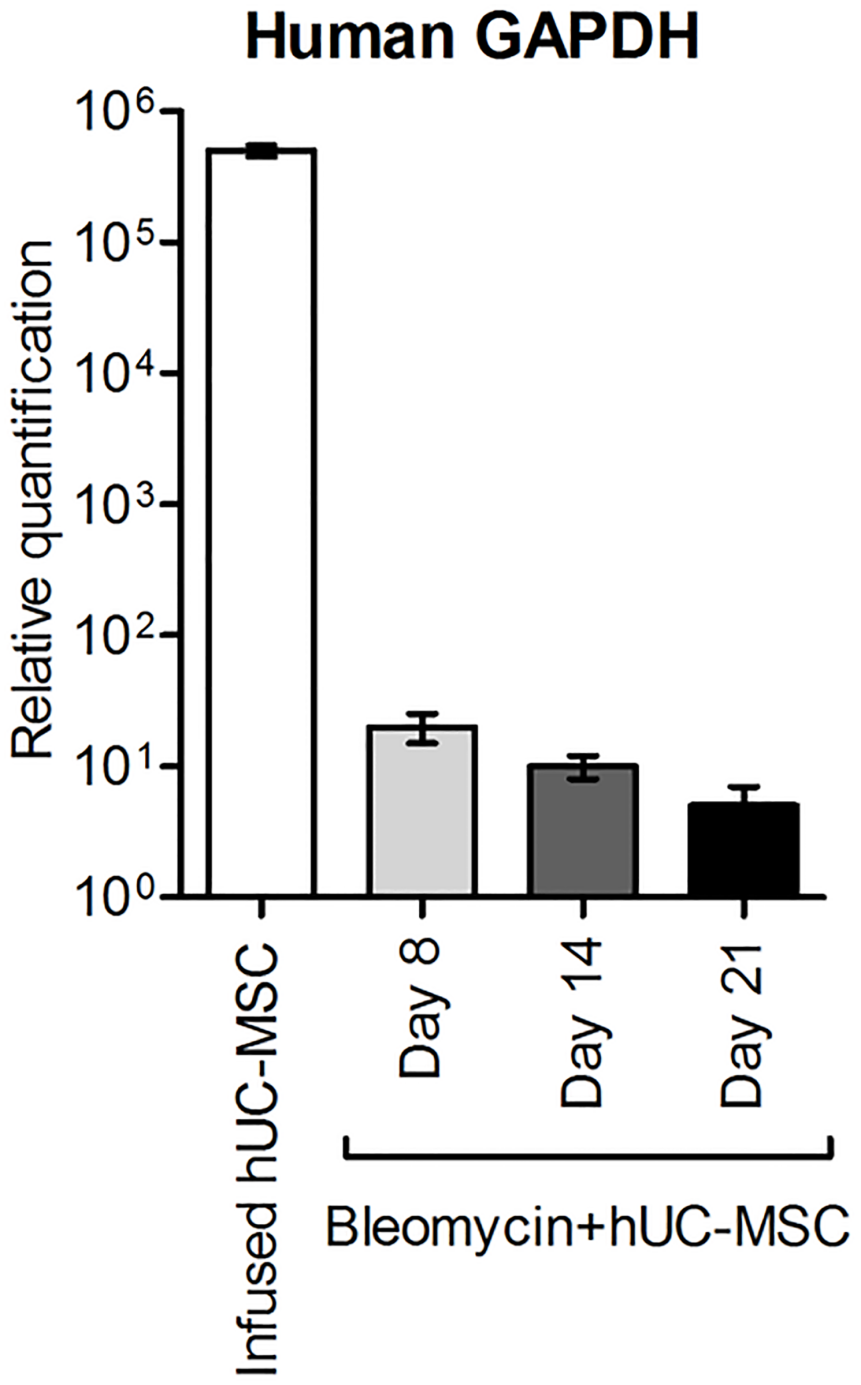
Detection of hUC-MSC by quantitative real-time PCR assay for human GAPDH. Human GAPDH assessed in RNA extracted from cultured hUC-MSC prior to infusion or from lung tissue of C57BL/6 mice (n = 5 per group) receiving endotracheal bleomycin followed by intravenous hUC-MSC (bleomycin+hUC-MSC). Days 8, 14, 21 refer to bleomycin administration, and correspond, respectively, to 1, 7 and 14 days after the second hUC-MSC infusion. Results are expressed as mean ± SD (n = 5 per group) and are representative of three independent experiments performed in triplicate.

**Fig 12:**
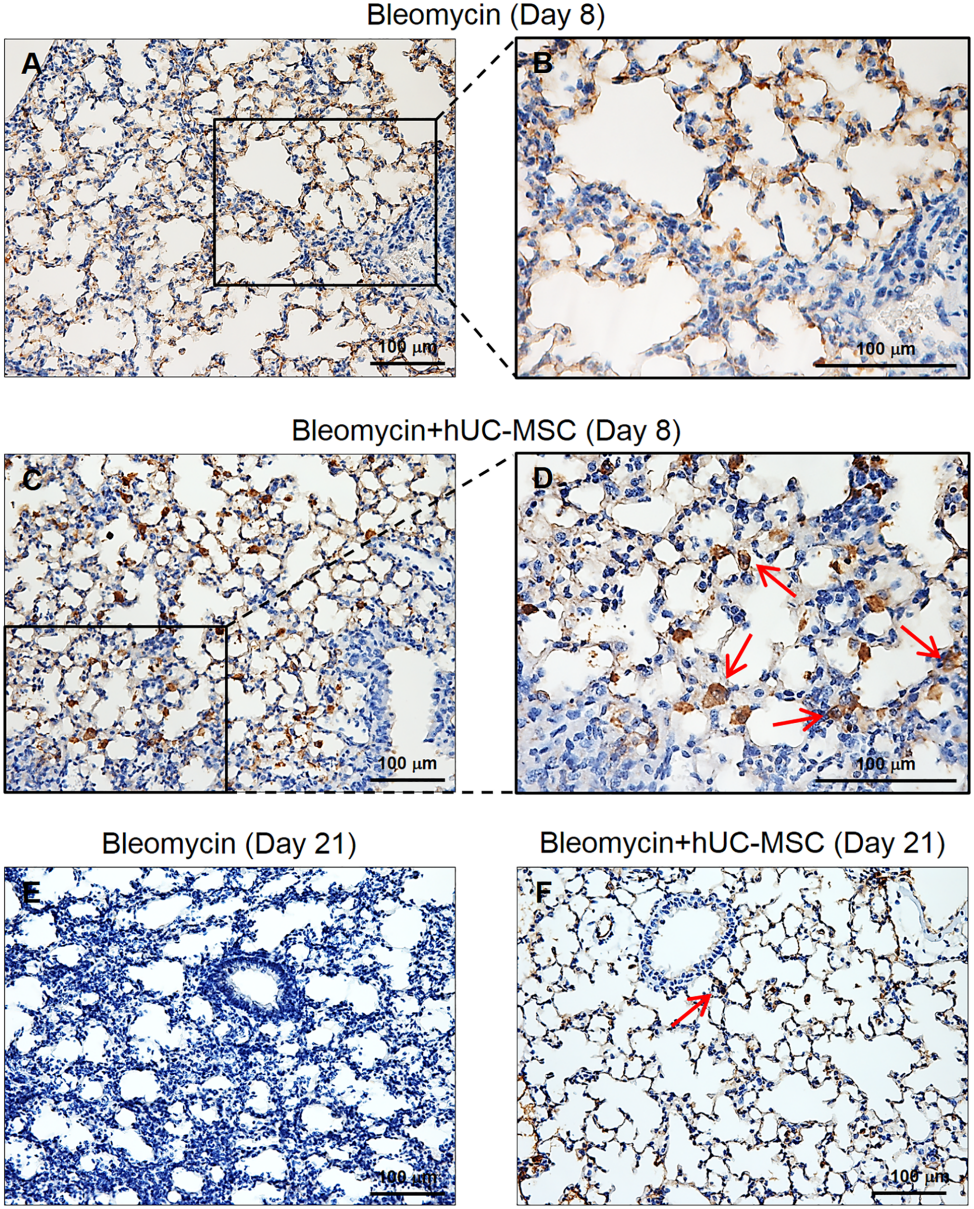
Detection of hUC-MSC in lung tissue by vimentin IHC. Lung sections obtained from C57BL/6 mice (n = 8 per group) receiving endotracheal bleomycin only (**A**, **B**, **E**) or endotracheal bleomycin followed by intravenous hUC-MSC (**C**, **D**, **F**) were immunostained with anti-human vimentin antibody. (**C,D)** Lung sections from hUC-MSC-treated mice show at day 8 (i.e. one day after second hUC-MSC infusion) numerous immunoreactive cells, distributed mainly in the septa and in the peribronchial tissue (200X), that exhibit at higher magnification (400X) a polygonal or stellate shape and enlarged nuclei (arrows). (**A,B**) In lung sections from mice treated with bleomycin only, the anti-human vimentin staining is extracellular and unspecific; no cells with polygonal or stellate shape reactive for human vimentin are evident (200X and 400X). (**F**) Lung sections from hUC-MSC-treated mice show at day 21 (i.e. 14 days after second hUC-MSC infusion) normal alveolar architecture with no fibrosis or inflammation, which are evident in the untreated mice (**E**), and only few vimentin-positive cells (arrows).

**Fig 13:**
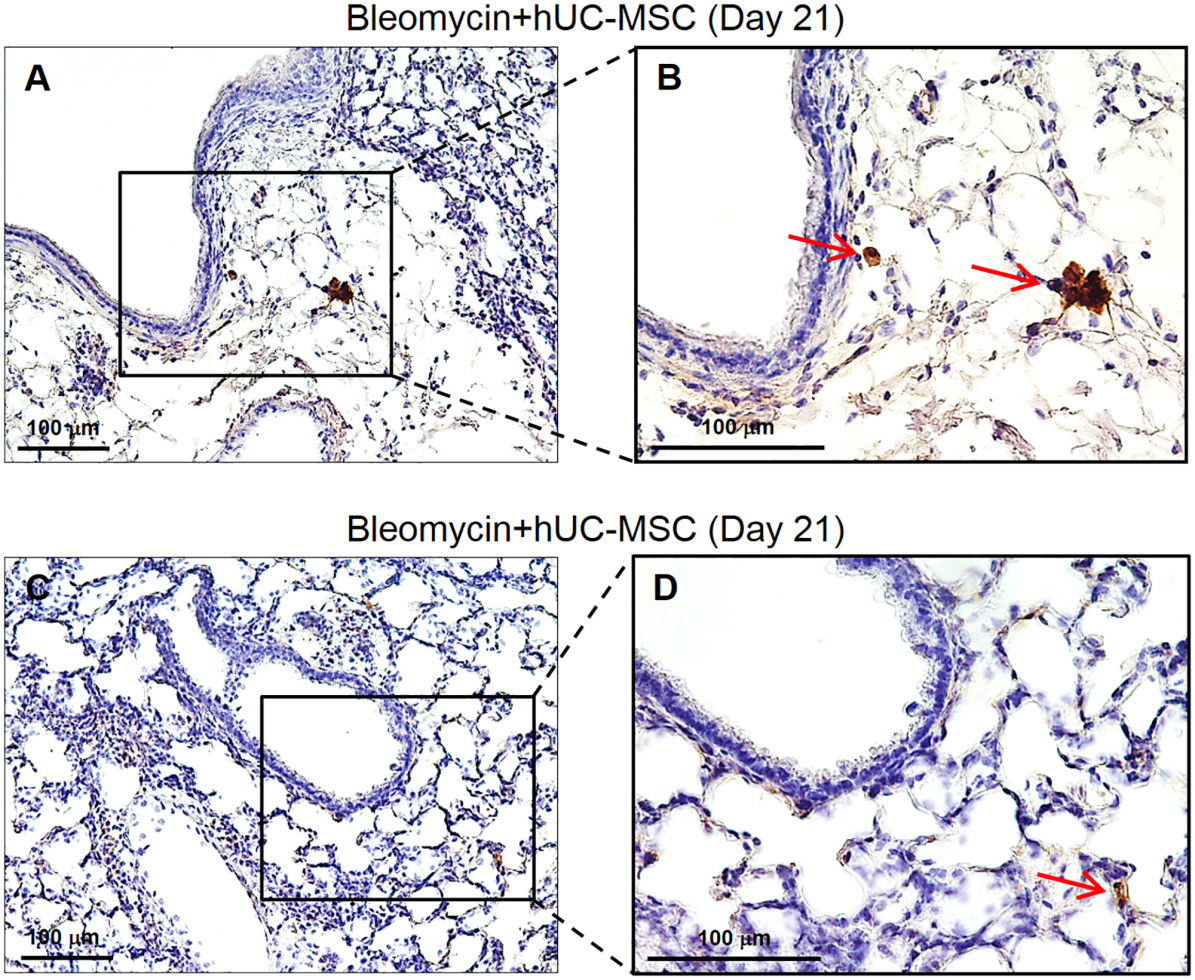
Detection of hUC-MSC in lung tissue by HLA-1 and CD105 IHC. Lung sections obtained from C57BL/6 mice receiving endotracheal bleomycin followed by intravenous hUC-MSC were immunostained with anti-HLA-1 (**A-B**) or anti-CD105 (**C-D**) antibodies. Representative microscopic images (200X and 400X) of three independent experiments are shown,. Lung sections from hUC-MSC-treated mice show at day 21 (i.e. 14 days after second hUC-MSC infusion) normal alveolar architecture with no fibrosis or inflammation, and only few positive cells (arrows, 400X).

To elucidate the kinetics of hUC-MSC engraftment into mouse lungs after the second i.v. injection, we sampled mice at days 8, 14 and 21 after bleomycin injury, i.e. 1, 7 and 14 days after the second hUC-MSC infusion in our experimental conditions (Fig 1). In order to employ a more sensitive detection method than IHC, we first amplified human GAPDH by quantitative real-time PCR in total mRNA from lung tissue. Of note, the source of human GAPDH transcript in our experimental setting can be provided exclusively by the hUC-MSC infused i.v. Results indicated the presence of low amount of this human transcript at each time point, with a decreasing trend from day 8 to day 21 (Fig 11). Then, based on previously published papers[34–36] reporting the use of anti-human vimentin antibody to mark hUC-MSC, we applied this IHC method for the staining of lung sections obtained at day 8 and day 21 both from mice treated with bleomycin only and from mice treated with bleomycin and hUC-MSC. The results (Fig 12) indicated that anti-human vimentin antibody, besides producing some extracellular cross-reactive background signal, mark a cell subtype that is present exclusively in lung sections from hUC-MSC-treated mice, with morphological characteristics (spindle, polygonal or stellate shape with abundant cytoplasm and enlarged nuclei) clearly different from fibroblasts or macrophages and with distribution in the septa and peribronchial tissue at inflammatory sites. These cells were still present at day 21, although reduced in number. Finally, to further confirm that these vimentin-positive cells are hUC-MSC, lung sections were immunolabeled with anti-human CD105 and anti-HLA-1 antibodies, which also showed similar positive cells (Fig 13). Taken together, our results are consistent with previous reports[18, 37–39] indicating homing of MSC in mouse lungs within the first 24 hours from systemic intravenous administration, followed by a fast dislodgement afterwards.

## Discussion

The results described herein provide further evidence supporting the use of hUC-MSC in the therapy of ILD caused by several different etiologies. In fact, the bleomycin-induced lung fibrosis mouse model is representative of many different disorders, ranging from idiopathic pulmonary fibrosis to connective tissue diseases. Within this broad spectrum, a shared feature is represented by inflammatory pathways that can be recapitulated in this experimental model. It was very interesting to find that the anti-inflammatory, anti-fibrotic effect of hUC-MSC infusion in these mice was not accompanied, at different sacrifice time points (8, 14 and 21 days post-injury, i.e. 1, 7 and 14 days after the second hUC-MSC administration), by a global decrease in pro-inflammatory cytokine production, but by a rather selective decrease of pro-inflammatory IL-6 levels. The significant reduction of IL-6 in hUC-MSC-treated mice co-occurred with a consistent decrease of IL-10 and TGFβ levels, although TGFβ changes were observed at later time points (days 14 and 21). IL-6 has been shown to induce activation of M2 macrophages and potentiation of their pro-fibrotic phenotype[33], which suggested M2 macrophages as possible cellular target of hUC-MSC biological activity in vivo. To verify this hypothesis, since IL-6 and also IL-10 can be also secreted in abundance by M1 macrophages and significant overlap between M1 and M2 macrophage phenotype can occur in many instances, general macrophage markers of chronic inflammation and fibrosis during bleomycin-induced fibrosis such as Galectin-3 [32] and more specific markers of M2 macrophage activation such as Arginase-1[33] were independently assessed in lung tissue after bleomycin injury with or without subsequent hUC-MSC infusion. Very similar patterns and counts of Galectin-3 and Arginase-1 positively stained cells were obtained, likely indicating M2 polarization of the macrophage population labeled by these two markers in our experimental setting.

In absence of hUC-MSC treatment, we could observe a significant increase in M2 macrophage count in the alveoli and interstitial spaces at day 8 and 21 post-bleomycin endotracheal injection, which was significantly diminished, although not completely abolished, at each time point by hUC-MSC infused at day 1 and 7 after bleomycin injury. Thus, our findings suggest a role of excessive proliferation and activation of M2 macrophages in the pathogenesis of lung fibrosis as a consequence of pro-inflammatory stimuli, and indicate that a potential therapeutic effect of hUC-MSC in vivo may reside in the ability of modulating the expansion and activity of M2 macrophages in response to inflammatory triggers. Consistently, in a different mouse model of lung fibrosis (hypochlorite-induced) treated with mouse bone marrow-derived MSC[40], amelioration of fibrosis at day 21 was associated with downregulation of the IL6-IL10-TGFβ axis, further corroborating the hypothesis of a critical involvement of M2 macrophages in lung fibrosis, and the ability of MSC in downregulating those inflammatory stimuli leading to persistent M2 activation and expansion. In point of fact, the potential detrimental role of M2 has already been postulated[41]. If the initial insult persists, chronic activation of M2 may lead to exacerbation of fibrosis through the release of TGF-β and other factors that stimulate resident fibroblasts to acquire a proliferative, synthetic, myofibroblast-like phenotype[42]. It must be noted that our study did not differentiate the total lung macrophage population from recruited circulating monocytes, which have been shown to be a major player in lung fibrosis[43] and therefore could also be the target of hUC-MSC.

The mechanism whereby hUC-MSC exert downregulation of the IL-6/IL-10/TGFβ axis involving lung M2 macrophages in the bleomycin mouse model, rather than a mere anti-inflammatory effect, is not immediately understandable in our experimental setting. However, tracing the engraftment of hUC-MSC in the bleomycin-injured lungs indicated homing of infused cells at inflammatory sites of mouse lungs within the first 24 hours from systemic intravenous administration. At this time point (day 8), a prominent IL-6 increase induced by bleomycin could be observed, which was significantly counteracted by hUC-MSC, whereas TGFβ was found only slightly higher than negative control. This corroborates the hypothesis of a rather selective downregulation of IL-6-secreting cells operated by hUC-MSC. Conversely, at later time points (1–2 weeks after i.v. infusion) fewer hUC-MSC could be detected. This time course is consistent with previous reports indicating a fast dislodgement of hUC-MSC from inflamed tissue (18,[21] 29, 32, [39, 44]. Alike, human adipose-derived MSC were shown to attenuate early stage bleomycin injury with similar kinetics[45]. Therefore, the suppressive effect of hUC-MSC on IL-6 mRNA transcription may be explained with the release of inhibitory factors such as TSG-6, a powerful modulator of inflammation secreted by hUC-MSC themselves[21], during their passage across the inflamed lung tissues. Indeed, all of the hUC-MSC employed in this study were TSG-6 positive prior to infusion in mice. Thus, it is conceivable that hUC-MSC may have transiently secreted this paracrine factor in response to proinflammatory cytokines in bleomycin-injured lungs upon each i.v. infusion, thereby attenuating the bleomycin-induced acute lung injury, the subsequent expansion of activated resident tissue M2 macrophages, and the resulting chronic stimulation of resident fibroblasts eventually leading to excessive deposition of extracellular matrix, as reported in other mouse models of chemical-induced inflammation[46]. The persistence of this biological effect, despite the transient presence of hUC-MSC, is consistent with previous reports involving lungs[44] and other organs[47]. It has been suggested by others[48] that short-term effects of MSC are mediated by their secretome, i.e. paracrine release of trophic factors by, or induced by, MSC, whereas their long-term effects are due to interaction and activation of other cell types in a probable “hit-and-run” way of action. Several studies have reported that the modulation of T-cell responses occur indirectly via MSC-mediated induction of regulatory T-cells (Tregs)[49]. Alternative mechanisms underlying the therapeutic effects of MSC engraftment into bleomycin-injured lungs, such as MSC differentiation into alveolar epithelial cells[50] have been proposed. Therefore, MSC seem to have the ability to induce sustained beneficial effects despite their disappearance from tissues.

Finally, the remarkable immunomodulatory effect of systemic administration of hUC-MSC was specific, as demonstrated by total inefficacy of human dermal fibroblasts in counteracting bleomycin injury, and targeted to bleomycin-injured lungs, as shown by the absence of any immunogenic activity in control mice receiving endotracheal saline solution. Moreover, no immune-mediated side effects such as fever, joint swelling, skin rash, or any other suffering manifestations were observed in fully immunocompetent mice like those employed in this study. Overall, the features of hUC-MSC described in this study provide proof of concept for testing the efficacy of this cell therapy in human diseases characterized by prominent lung fibrosis, such as SSc-ILD. This will require the design of adequate clinical trials in appropriate cohorts of patients, for instance immunosuppressant-naïve SSc patients with early lung involvement. Alternatively, IPF patients may also benefit from this cellular therapy, as suggested by a recent case report[51]. This clinical case indicated that hUC-MSC do not have to be exactly matched with the recipient to avoid a significant graft-versus-host reaction, which emphasizes the feasibility of such therapeutic approach. Finally, since inflammation and fibrosis are predominant features of the aging lung[52], it is conceivable that hUC-MSC may be tested in mouse models of accelerated senescence[53] to check their efficacy in counteracting detrimental phenomena correlated to physiological and/or pathological aging. Another potential field of application may be represented by diseases natively non-fibrotic such as chronic obstructive pulmonary disease (COPD)[54], also characterized by inflammation and fibrosis in later or therapy-refractory stages.

## Supporting information

S1 File Authorization to hUC-MSC isolation by ethics committee of Papa Giovanni XXIII Hospital, Bergamo, Italy. (PDF)
