# Supplementary material for: Mesenchymal stromal cells from human umbilical cord prevent the development of lung fibrosis in immunocompetent mice
Source: PLoS One. 2018 Jun 1;13(6):e0196048. doi: 10.1371/journal.pone.0196048 (PMC5983506; doi:10.1371/journal.pone.0196048)
Supplement: S1 File — (PDF) [file pone.0196048.s001.pdf]

Sistema Socio Sanitario

Ospedale Papa Giovanni XXIII

Regione Lombardia

ASST Papa Giovanni XXIII

DELIBERAZIONE NR. 1239 DEL 13/07/2017

OGGETTO: AUTORIZZAZIONE ALL'AVVIO DELLO STUDIO "PHENOTYPICAL AND FUNCTIONAL CHARACTERIZATION OF MESENCHYMAL STROMA CELLS (MSC) ISOLATED FROM DIFFERENT SOURCES" (REG. 2017-0101) PRESSO L'UOS CENTRO DI TERAPIA CELLULARE DI CUI È PROMOTORE ASST PAPA GIOVANNI XXIII.

IL DIRETTORE GENERALE
nella persona del Dott. Carlo Nicora

ASSISTITO DA:

IL DIRETTORE AMMINISTRATIVO	DR. VINCENZO PETRONELLA
IL DIRETTORE SANITARIO	DOTT. FABIO PEZZOLI
IL DIRETTORE SOCIO SANITARIO	DR.SSA DONATELLA VASATURO

Visti il d.lgs. 30.12.1992 n. 502 ed in particolare gli artt. 3 e 3bis e il d.lgs. 19.6.1999 n. 229;

Vista la l. 7.8.1990 n. 241 e s.m.i. “Nuove norme in materia di procedimento amministrativo e di diritto di accesso ai documenti amministrativi”;

Vista la l.r. 30.12.2009 n. 33, riguardante il testo unico delle l.r. in materia di sanità, come modificata dalla l.r. 11 agosto 2015 n. 23 “Evoluzione del sistema sociosanitario lombardo: modifiche al titolo I e II della legge regionale 30.12.2009 n. 33”;

Vista la deliberazione di Giunta regionale n. X/4487 del 10.12.2015, in attuazione della su richiamata l.r. 11.8.2015 n. 23, con la quale è stata costituita a partire dall’1.1.2016 l’azienda socio - sanitaria territoriale (ASST) Papa Giovanni XXIII con sede legale in Piazza OMS 1 – 24127 Bergamo - nel nuovo assetto indicato dall’allegato 1 al medesimo provvedimento;

Vista la deliberazione di Giunta regionale n. X/4644 del 19.12.2015 con la quale è stato nominato direttore generale dell’ASST Papa Giovanni XXIII con decorrenza dall’1.1.2016 e fino al 31.12.2018;

Vista la deliberazione n. 1 del 4.1.2016 “Presa d’atto della deliberazione di Giunta regionale n. X/4644 del 19.12.2015 di nomina del direttore generale dell’azienda socio - sanitaria territoriale Papa Giovanni XXIII di Bergamo. Relativo insediamento”;

Rilevato che il responsabile del procedimento riferisce quanto segue:

- il responsabile dell'UOS Centro di terapia cellulare ha proposto la conduzione presso la propria UOS dello studio "Phenotypical and functional characterization of mesenchymal stroma cells (MSC) isolated from different sources" (reg. 2017-0101), su tessuti di donatori di midollo osseo, di donatrici del funicolo cordonale, di pazienti affetti da malattie ematologiche;
- trattasi di studio no-profit con validità fino a dicembre 2022, senza oneri aggiuntivi per l'azienda e senza la ripartizione di alcun compenso tra quanti collaborano allo stesso;
- con scritto ricevuto in data 24/05/2017, il dott. Martino Introna, responsabile dell'UOS Centro di terapia cellulare ha comunicato la previsione di arruolamento di n. 50 pazienti, proponendosi quale sperimentatore principale;
- il predetto responsabile ha fornito i dati necessari per la valutazione di fattibilità locale e ha prodotto la documentazione prevista dall'"Istruzione operativa: modalità di presentazione della documentazione al Comitato etico di Bergamo da parte del promotore e/o sperimentatore", dalla quale si rilevano le caratteristiche dello studio e alla quale si rinvia per gli eventuali approfondimenti;
- l'analisi di fattibilità locale per la valutazione di copertura dei costi a carico dell'azienda ha dato esito positivo;
- il Comitato etico di Bergamo ha espresso parere favorevole in data 09/06/2017;

Acquisito il parere del direttore amministrativo, del direttore sanitario e del direttore sociosanitario.

DELIBERA

1. di approvare le premesse al presente provvedimento di cui sono parte integrante;
2. di autorizzare l'avvio dello studio "Phenotypical and functional characterization of mesenchymal stroma cells (MSC) isolated from different sources" (reg. 2017-0101), su tessuti derivanti da donatori di midollo osseo, da donatrici del funicolo cordonale, da pazienti affetti da malattie ematologiche, presso l'UOS Centro di terapia cellulare;
3. di affidare la responsabilità di sperimentatore principale al dott. Martino Introna.

IL DIRETTORE GENERALE
Dott. Carlo Nicora

Il responsabile del procedimento: dott. Mario Fraticelli USC Formazione marketing e libera professione

Documento prodotto in originale informatico e firmato digitalmente dal direttore generale ai sensi del "Codice dell'amministrazione digitale" (d.lgs. n. 82/2005 e s.m.i.)

ATTESTAZIONE DI REGOLARITA' AMMINISTRATIVO-CONTABILE (proposta n. 395/2017)

Oggetto: AUTORIZZAZIONE ALL'AVVIO DELLO STUDIO "PHENOTYPICAL AND FUNCTIONAL CHARACTERIZATION OF MESENCHYMAL STROMA CELLS (MSC) ISOLATED FROM DIFFERENT SOURCES" (REG. 2017-0101) PRESSO L'UOS CENTRO DI TERAPIA CELLULARE DI CUI È PROMOTORE ASST PAPA GIOVANNI XXIII.

UOC PROPONENTE

Si attesta la regolarità tecnica del provvedimento, essendo state osservate le norme e le procedure previste per la specifica materia.

Si precisa, altresì, che:

A. il provvedimento:

☐ prevede
☒ non prevede

COSTI diretti a carico dell'ASST

B. il provvedimento:

☐ prevede
☒ non prevede

RICAVI da parte dell'ASST.

Bergamo, 28/06/2017

Il direttore

Dr. / Dr.ssa Fraticelli Mario

PARERE DIRETTORI

all’adozione della proposta di deliberazione N.395/2017
ad oggetto:
AUTORIZZAZIONE ALL'AVVIO DELLO STUDIO "PHENOTYPICAL AND FUNCTIONAL CHARACTERIZATION OF MESENCHYMAL STROMA CELLS (MSC) ISOLATED FROM DIFFERENT SOURCES" (REG. 2017-0101) PRESSO L'UOS CENTRO DI TERAPIA CELLULARE DI CUI È PROMOTORE ASST PAPA GIOVANNI XXIII.

Ciascuno per gli aspetti di propria competenza, vista anche l’attestazione di regolarità amministrativo-contabile.

**DIRETTORE AMMINISTRATIVO :**<br>Ha espresso il seguente parere:<br>☒ FAVOREVOLE<br>☐ NON FAVOREVOLE<br>☐ ASTENUTO	Petronella Vincenzo
Note:	

**DIRETTORE SANITARIO :**<br>Ha espresso il seguente parere:<br>☒ FAVOREVOLE<br>☐ NON FAVOREVOLE<br>☐ ASTENUTO	Pezzoli Fabio
Note:	

**DIRETTORE SOCIOSANITARIO :**<br>Ha espresso il seguente parere:<br>☒ FAVOREVOLE<br>☐ NON FAVOREVOLE<br>☐ ASTENUTO	Vasaturo Donatella
Note:	

CERTIFICATO DI PUBBLICAZIONE

Pubblicata all’Albo Pretorio on-line
dell’Azienda socio sanitaria territoriale

“Papa Giovanni XXIII” Bergamo

per 15 giorni

--
